# 

**Published:** 1881-09

**Authors:** 


HERMAN MYNTER, M. D.

### CONTENTS:

INDEX MEDICUS.

| | PAGE. |
|---|---|
| **ORIGINAL COMMUNICATIONS:** | |
| Decision of the Hon. George Parker, Judge of the Supreme Court, Regarding the Legality of the Charter of the College of Physicians and Surgeons of Buffalo, | 49 |
| The Relation of Diseases. By P. W. Van Peyma, M. D., | 60 |
| **CLINICAL REPORTS:** | |
| Urethrotomia Externa. By Herman Mynter, M. D., | 67 |
| **TRANSLATIONS:** | |
| A Successful Case of Resection of the Bowels. Reported by Dr. Roggenbon from Prof. Treudelenburg's Clinic in Rostock. Translated from the German, | 69 |
| **SELECTIONS:** | |
| Rapid Breathing. By Addinell Hewson, Senior, A. M., M. D., | 72 |
| Female Doctors, | 77 |
| Cremation, | 79 |
| Remarkable Case of Early Maternity, | 81 |
| "Fooled by Temperature." | 82 |
| Discovery of the Micrococcus of Syphilis, | 83 |
| Quack Advertisements, | 84 |
| Antiseptic Osteotomy of the Tibia; Rapidly Fatal Carbolic Intoxication. | 85 |
| Special Report on the International Medical and Sanitary Exhibition, South Kensington, | 88 |
| **EDITORIAL:** | |
| Buffalo College of Physicians and Surgeons, | 89 |
| International Medical Congress, | 91 |
| Letter of A. M. Barker, M. D., Secretary, | 93 |
| **REVIEWS,** | 94 |

